# 4-Meth­oxy-3-(meth­oxy­meth­yl)benzalde­hyde

**DOI:** 10.1107/S1600536812050350

**Published:** 2012-12-19

**Authors:** Jing-Chao Zhang, Jun Sun, Juan Zhang, Guang-Lin Liu, Cheng Guo

**Affiliations:** a Department of Applied Chemistry, College of Science, Nanjing University of Technology, Nanjing 211816, People’s Republic of China

## Abstract

In the title compound, C_10_H_12_O_3_, the dihedral angle between the benzene ring and the meth­oxy­methyl side chain is 9.7 (2)°. The O atom of the aldehyde group and the C atom of the meth­oxy group deviate from the plane of the ring by 0.039 (3) and 0.338 (4) Å, respectively. The only inter­molecular inter­actions are very weak C—H⋯π inter­actions.

## Related literature

For the synthesis and applications of the title compound see: Jonali *et al.* (2003 ▶).
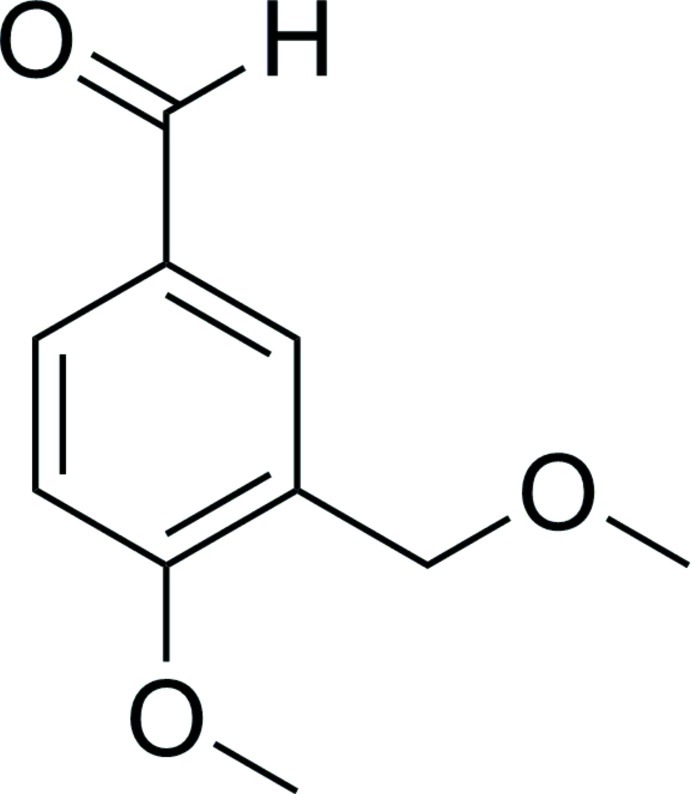


## Experimental

### 

#### Crystal data

C_10_H_12_O_3_
*M* *_r_* = 180.20
Monoclinic, 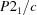

*a* = 7.8100 (16) Å
*b* = 8.3970 (17) Å
*c* = 14.510 (3) Å
β = 98.01 (3)°
*V* = 942.3 (3) Å^3^
*Z* = 4
Mo *K*α radiation
μ = 0.09 mm^−1^
*T* = 293 K
0.30 × 0.20 × 0.10 mm

#### Data collection

Enraf–Nonius CAD-4 diffractometer
Absorption correction: ψ scan (North *et al.*, 1968 ▶) *T*_min_ = 0.973, *T*_max_ = 0.991
1860 measured reflections
1729 independent reflections
860 reflections with *I* > 2σ(*I*)
*R*_int_ = 0.099
3 standard reflections every 200 reflections intensity decay: 1%

#### Refinement

*R*[*F* ^2^ > 2σ(*F* ^2^)] = 0.063
*wR*(*F* ^2^) = 0.165
*S* = 1.01
1729 reflections
118 parameters
H-atom parameters constrained
Δρ_max_ = 0.19 e Å^−3^
Δρ_min_ = −0.18 e Å^−3^

### 

Data collection: *CAD-4 Software* (Enraf–Nonius, 1985 ▶); cell refinement: *CAD-4 Software*; data reduction: *XCAD4* (Harms & Wocadlo, 1995 ▶); program(s) used to solve structure: *SHELXS97* (Sheldrick, 2008 ▶); program(s) used to refine structure: *SHELXL97* (Sheldrick, 2008 ▶); molecular graphics: *SHELXTL* (Sheldrick, 2008 ▶); software used to prepare material for publication: *SHELXTL*.

## Supplementary Material

Crystal structure: contains datablock(s) I, global. DOI: 10.1107/S1600536812050350/hb7010sup1.cif

Structure factors: contains datablock(s) I. DOI: 10.1107/S1600536812050350/hb7010Isup3.hkl

Supplementary material file. DOI: 10.1107/S1600536812050350/hb7010Isup3.cml

Additional supplementary materials:  crystallographic information; 3D view; checkCIF report

## Figures and Tables

**Table 1:** Hydrogen-bond geometry (Å, °) *Cg*1 is the centroid of the C1–C6 ring.

*D*—H⋯*A*	*D*—H	H⋯*A*	*D*⋯*A*	*D*—H⋯*A*
C7—H7*B*⋯*Cg*1^i^	0.96	2.84	3.650 (5)	143
C8—H8*A*⋯*Cg*1^ii^	0.97	2.97	3.731 (3)	136

Symmetry codes: (i) 
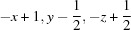
; (ii) 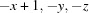.

**Fig. 1.**
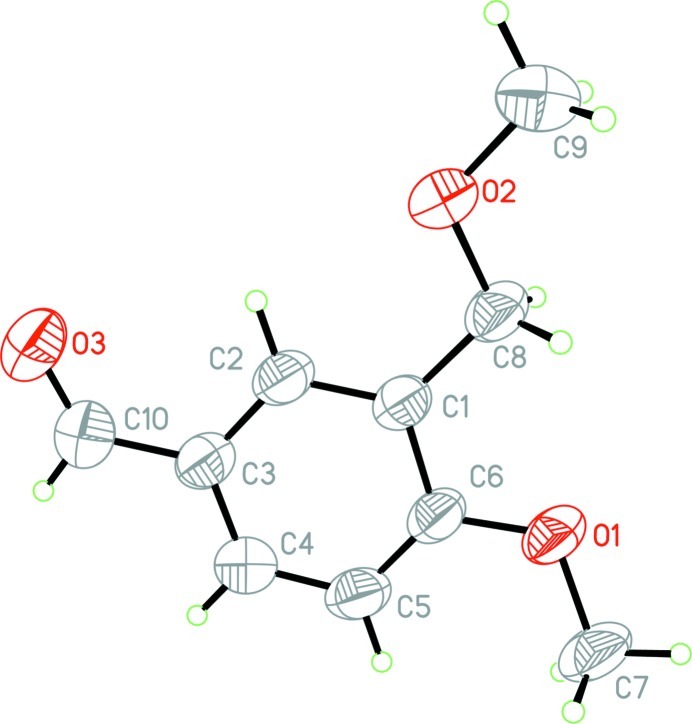
The molecular structure of (I), with displacement ellipsoids drawn at the 50% probability level.

**Fig. 2.**
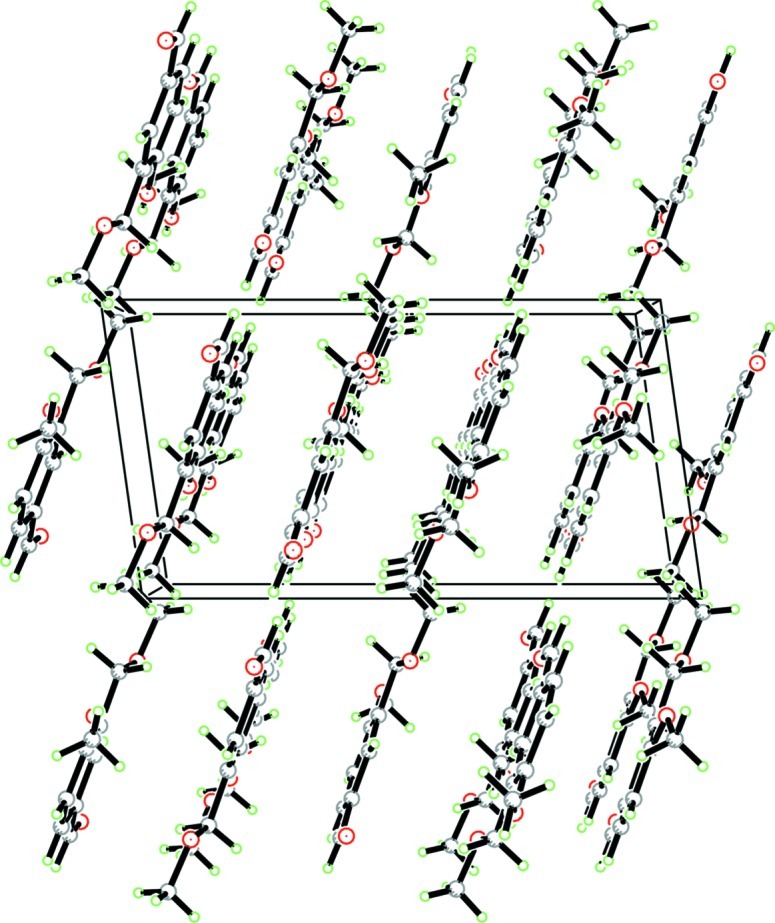
A packing diagram of (I).

## supplementary crystallographic information

### Experimental

The title compound, (I) was prepared by the literature method (Jonali *et al.* 2003). Colourless blocks were obtained by dissolving (I) (0.18 g, 1.0 mmol) in acetone (25 ml) and evaporating the solvent slowly at room temperature for about 7 d.

### Refinement

H atoms were positioned geometrically and refined as riding groups with *U*_iso_(H) = *xU*_eq_(C), where *x* = 1.2 for aromatic H, and *x* = 1.5 for other H.

### Crystal data

**Table:**

C_10_H_12_O_3_	*F*(000) = 384
*M**_r_* = 180.20	*D*_x_ = 1.270 Mg m^−^^3^
Monoclinic, *P*2_1_/*c*	Melting point: 341 K
Hall symbol: -P 2ybc	Mo *K*α radiation, λ = 0.71073 Å
*a* = 7.8100 (16) Å	Cell parameters from 25 reflections
*b* = 8.3970 (17) Å	θ = 9–13°
*c* = 14.510 (3) Å	µ = 0.09 mm^−^^1^
β = 98.01 (3)°	*T* = 293 K
*V* = 942.3 (3) Å^3^	Block, colourless
*Z* = 4	0.30 × 0.20 × 0.10 mm

### Data collection

**Table:**

Enraf–Nonius CAD-4 diffractometer	860 reflections with *I* > 2σ(*I*)
Radiation source: fine-focus sealed tube	*R*_int_ = 0.099
Graphite monochromator	θ_max_ = 25.4°, θ_min_ = 2.6°
ω/2θ scans	*h* = 0→9
Absorption correction: ψ scan (North *et al.*, 1968)	*k* = 0→10
*T*_min_ = 0.973, *T*_max_ = 0.991	*l* = −17→17
1860 measured reflections	3 standard reflections every 200 reflections
1729 independent reflections	intensity decay: 1%

### Refinement

**Table:**

Refinement on *F*^2^	Primary atom site location: structure-invariant direct methods
Least-squares matrix: full	Secondary atom site location: difference Fourier map
*R*[*F*^2^ > 2σ(*F*^2^)] = 0.063	Hydrogen site location: inferred from neighbouring sites
*wR*(*F*^2^) = 0.165	H-atom parameters constrained
*S* = 1.01	*w* = 1/[σ^2^(*F*_o_^2^) + (0.070*P*)^2^] where *P* = (*F*_o_^2^ + 2*F*_c_^2^)/3
1729 reflections	(Δ/σ)_max_ < 0.001
118 parameters	Δρ_max_ = 0.19 e Å^−^^3^
0 restraints	Δρ_min_ = −0.18 e Å^−^^3^

### Special details

**Table:**

Geometry. All e.s.d.'s (except the e.s.d. in the dihedral angle between two l.s. planes) are estimated using the full covariance matrix. The cell e.s.d.'s are taken into account individually in the estimation of e.s.d.'s in distances, angles and torsion angles; correlations between e.s.d.'s in cell parameters are only used when they are defined by crystal symmetry. An approximate (isotropic) treatment of cell e.s.d.'s is used for estimating e.s.d.'s involving l.s. planes.
Refinement. Refinement of *F*^2^ against ALL reflections. The weighted *R*-factor *wR* and goodness of fit *S* are based on *F*^2^, conventional *R*-factors *R* are based on *F*, with *F* set to zero for negative *F*^2^. The threshold expression of *F*^2^ > σ(*F*^2^) is used only for calculating *R*-factors(gt) *etc*. and is not relevant to the choice of reflections for refinement. *R*-factors based on *F*^2^ are statistically about twice as large as those based on *F*, and *R*- factors based on ALL data will be even larger.

### Fractional atomic coordinates and isotropic or equivalent isotropic displacement parameters (Å^2^)

**Table:**

	*x*	*y*	*z*	*U*_iso_*/*U*_eq_	
O1	0.3742 (3)	−0.2060 (2)	0.10208 (17)	0.0606 (7)	
C1	0.4323 (4)	0.0653 (3)	0.1125 (2)	0.0449 (8)	
O2	0.2282 (3)	0.2507 (3)	0.03860 (18)	0.0736 (8)	
C2	0.5453 (4)	0.1885 (4)	0.1380 (2)	0.0512 (9)	
H2A	0.5092	0.2927	0.1250	0.061*	
O3	0.8048 (3)	0.4313 (3)	0.1968 (2)	0.0838 (10)	
C3	0.7125 (5)	0.1612 (4)	0.1829 (2)	0.0531 (9)	
C4	0.7648 (4)	0.0059 (4)	0.2027 (3)	0.0608 (10)	
H4A	0.8752	−0.0133	0.2339	0.073*	
C5	0.6561 (5)	−0.1209 (4)	0.1772 (3)	0.0579 (10)	
H5A	0.6928	−0.2248	0.1904	0.070*	
C6	0.4919 (4)	−0.0912 (3)	0.1318 (2)	0.0494 (9)	
C7	0.4335 (5)	−0.3667 (4)	0.1007 (3)	0.0688 (12)	
H7A	0.3380	−0.4353	0.0791	0.103*	
H7B	0.4826	−0.3978	0.1624	0.103*	
H7C	0.5196	−0.3749	0.0598	0.103*	
C8	0.2523 (4)	0.0900 (3)	0.0654 (2)	0.0547 (9)	
H8A	0.2307	0.0221	0.0110	0.066*	
H8B	0.1709	0.0614	0.1075	0.066*	
C9	0.0623 (5)	0.2781 (4)	−0.0121 (3)	0.0835 (13)	
H9A	0.0509	0.3887	−0.0286	0.125*	
H9B	−0.0248	0.2497	0.0256	0.125*	
H9C	0.0485	0.2145	−0.0676	0.125*	
C10	0.8314 (5)	0.2919 (5)	0.2107 (3)	0.0680 (11)	
H10A	0.9390	0.2644	0.2425	0.082*	

### Atomic displacement parameters (Å^2^)

**Table:**

	*U*^11^	*U*^22^	*U*^33^	*U*^12^	*U*^13^	*U*^23^
O1	0.0666 (16)	0.0309 (13)	0.0856 (18)	−0.0025 (11)	0.0151 (13)	−0.0033 (11)
C1	0.050 (2)	0.0358 (18)	0.052 (2)	−0.0002 (16)	0.0158 (16)	−0.0018 (14)
O2	0.0662 (17)	0.0371 (14)	0.112 (2)	0.0032 (13)	−0.0070 (15)	0.0046 (13)
C2	0.058 (2)	0.0342 (17)	0.063 (2)	0.0024 (16)	0.0133 (18)	0.0005 (15)
O3	0.082 (2)	0.0481 (16)	0.118 (3)	−0.0127 (14)	0.0024 (17)	−0.0007 (16)
C3	0.058 (2)	0.0404 (19)	0.062 (2)	0.0006 (18)	0.0120 (18)	−0.0036 (17)
C4	0.055 (2)	0.053 (2)	0.075 (3)	0.0049 (19)	0.0107 (19)	−0.0041 (19)
C5	0.064 (2)	0.040 (2)	0.073 (3)	0.0082 (18)	0.019 (2)	0.0009 (18)
C6	0.057 (2)	0.0321 (18)	0.061 (2)	−0.0024 (16)	0.0157 (19)	−0.0034 (15)
C7	0.084 (3)	0.0297 (18)	0.097 (3)	0.0005 (18)	0.029 (2)	−0.0046 (18)
C8	0.060 (2)	0.0332 (18)	0.072 (3)	−0.0042 (16)	0.0143 (19)	0.0049 (16)
C9	0.066 (3)	0.062 (3)	0.119 (4)	0.016 (2)	0.000 (3)	0.004 (2)
C10	0.060 (2)	0.058 (2)	0.084 (3)	−0.007 (2)	0.006 (2)	−0.003 (2)

### Geometric parameters (Å, º)

**Table:**

O1—C6	1.360 (4)	C4—H4A	0.9300
O1—C7	1.427 (3)	C5—C6	1.380 (4)
C1—C2	1.376 (4)	C5—H5A	0.9300
C1—C6	1.409 (4)	C7—H7A	0.9600
C1—C8	1.490 (4)	C7—H7B	0.9600
O2—C8	1.410 (3)	C7—H7C	0.9600
O2—C9	1.416 (4)	C8—H8A	0.9700
C2—C3	1.395 (5)	C8—H8B	0.9700
C2—H2A	0.9300	C9—H9A	0.9600
O3—C10	1.201 (4)	C9—H9B	0.9600
C3—C4	1.385 (4)	C9—H9C	0.9600
C3—C10	1.457 (5)	C10—H10A	0.9300
C4—C5	1.380 (4)		
			
C6—O1—C7	118.0 (3)	O1—C7—H7B	109.5
C2—C1—C6	117.8 (3)	H7A—C7—H7B	109.5
C2—C1—C8	123.2 (3)	O1—C7—H7C	109.5
C6—C1—C8	119.0 (3)	H7A—C7—H7C	109.5
C8—O2—C9	112.1 (3)	H7B—C7—H7C	109.5
C1—C2—C3	121.7 (3)	O2—C8—C1	109.9 (3)
C1—C2—H2A	119.2	O2—C8—H8A	109.7
C3—C2—H2A	119.2	C1—C8—H8A	109.7
C4—C3—C2	118.8 (3)	O2—C8—H8B	109.7
C4—C3—C10	119.6 (3)	C1—C8—H8B	109.7
C2—C3—C10	121.6 (3)	H8A—C8—H8B	108.2
C5—C4—C3	121.2 (3)	O2—C9—H9A	109.5
C5—C4—H4A	119.4	O2—C9—H9B	109.5
C3—C4—H4A	119.4	H9A—C9—H9B	109.5
C4—C5—C6	119.0 (3)	O2—C9—H9C	109.5
C4—C5—H5A	120.5	H9A—C9—H9C	109.5
C6—C5—H5A	120.5	H9B—C9—H9C	109.5
O1—C6—C5	124.4 (3)	O3—C10—C3	126.8 (4)
O1—C6—C1	114.1 (3)	O3—C10—H10A	116.6
C5—C6—C1	121.5 (3)	C3—C10—H10A	116.6
O1—C7—H7A	109.5		
			
C6—C1—C2—C3	−1.0 (5)	C4—C5—C6—C1	−1.0 (5)
C8—C1—C2—C3	179.3 (3)	C2—C1—C6—O1	−178.2 (3)
C1—C2—C3—C4	−0.6 (5)	C8—C1—C6—O1	1.5 (4)
C1—C2—C3—C10	−179.4 (3)	C2—C1—C6—C5	1.8 (5)
C2—C3—C4—C5	1.4 (5)	C8—C1—C6—C5	−178.4 (3)
C10—C3—C4—C5	−179.8 (3)	C9—O2—C8—C1	176.0 (3)
C3—C4—C5—C6	−0.6 (5)	C2—C1—C8—O2	9.5 (4)
C7—O1—C6—C5	−13.0 (5)	C6—C1—C8—O2	−170.2 (3)
C7—O1—C6—C1	167.1 (3)	C4—C3—C10—O3	178.7 (4)
C4—C5—C6—O1	179.0 (3)	C2—C3—C10—O3	−2.5 (6)

### Hydrogen-bond geometry (Å, º)

Cg1 is the centroid of the C1–C6 ring.

**Table:**

*D*—H···*A*	*D*—H	H···*A*	*D*···*A*	*D*—H···*A*
C7—H7*B*···*Cg*1^i^	0.96	2.84	3.650 (5)	143
C8—H8*A*···*Cg*1^ii^	0.97	2.97	3.731 (3)	136

Symmetry codes: (i) −*x*+1, *y*−1/2, −*z*+1/2; (ii) −*x*+1, −*y*, −*z*.
